# Lessons from the pilot of a mobile application to map assistive technology suppliers in Africa

**DOI:** 10.4102/ajod.v7i0.422

**Published:** 2018-03-29

**Authors:** Surona J. Visagie, Rebecca Matter, George M. Kayange, Mussa Chiwaula, Mark Harniss, Gubela Mji, Elsje Scheffler

**Affiliations:** 1 Centre for Rehabilitation Studies, Stellenbosch University, South Africa; 2 School of Public Health and Family Medicine, University of Cape Town, South Africa; 3 International Program on Disability, Technology and Rehabilitation, University of Washington, United States; 4 Southern Africa Federation of the Disabled (SAFOD), Gaborone, Botswana; 5 Rehabilitation Medicine, University of Washington, United States

## Abstract

A pilot project to develop and implement a mobile smartphone application (App) that tracks and maps assistive technology (AT) availability in southern Africa was launched in Botswana in 2016. The App was developed and tested through an iterative process. The concept of the App (AT-Info-Map) was well received by most stakeholders within the pilot country, and broader networks.

Several technical and logistical obstacles were encountered. These included high data costs; difficulty in accessing AT information from the public healthcare sector, the largest supplier of AT; and the high human resource demand of collecting and keeping up-to-date device-level information within a complex and fragmented supply sector that spans private, public and civil society entities. The challenges were dealt with by keeping the data burden low and eliminating product-level tracking. The App design was expanded to include disability services, contextually specific AT categories and make navigation more intuitive.

Long-term sustainability strategies like generating funding through advertisements on the App or supplier usage fees must be explored. Outreach and sensitisation programmes about both the App and AT in general must be intensified. The project team must continually strengthen partnerships with private and public stakeholders to ensure ongoing project engagement. The lessons learnt might be of value to others who wish to embark on initiatives in AT and/or implement Apps in health or disability in southern Africa and in low-resourced settings around the world.

## Background

The unmet need for assistive technology (AT) in developing countries is around 85% and is growing because of the changing demographics of disease (Harniss, Raja & Matter 2008; Matter et al. 2017). In sub-Saharan Africa, critical issues for persons with disabilities^1^ are lack of information and knowledge on the availability of AT products and services, lack of funds and a shortage of service providers. These challenges result in low acquisition rates of AT (Matter et al. 2017), which affect function and community integration negatively [Borg, Larsson & Östergren 2011; World Health Organization (WHO) 2014].

The leadership of the Southern African Federation for the Disabled (SAFOD) determined a broad project concept – locating AT suppliers in southern Africa. This concept was crafted into a proposal to create a user-friendly mechanism to efficiently track and map AT availability, so that those seeking AT can easily locate what they need, and to illuminate the gaps in AT availability. In December 2015, the project was selected as one of 29 grantees in the Google Impact Challenge – Disabilities (https://www.google.org/impactchallenge/disabilities/) and funded for a 3-year period (March 2016–April 2019).

CommCare (www.commcarehq.org/solutions/) based on the Open Data Kit was selected as the data platform to map AT availability because of its unique features designed for low-resourced settings such as offline use once installed. Dimagi (https://www.dimagi.com/) led the scoping and design process, with guidance from AT experts at the University of Washington, USA, and Stellenbosch University, South Africa, to ensure a design both accessible to users with different disabilities and aligned with global AT standards. SAFOD, the primary implementing partner, which includes 10 national federations representing disability organisations (DPOs) in member states, is based in Botswana where the pilot phase of the project was rolled out.

This case study describes the lessons learnt from the pilot phase, provides recommendations for scaling up of the AT-Info-Map to other SAFOD countries and might provide some guidance to others who wish to embark on similar projects in southern Africa and other low-resourced settings.

## Roll-out

**FIGURE 1:**
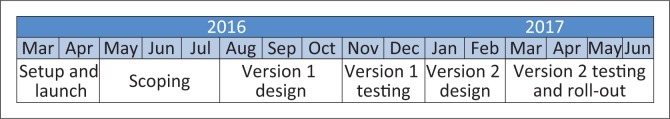
Timeline of the launch, development process and pilot roll-out.

The project was officially launched by SAFOD in Gaborone on 19 April 2016, after a preliminary meeting at Stellenbosch University in Cape Town (http://assistivetechmap.org/wp-content/uploads/2017/08/AT-Info-Map_CapeTown_summary_final.pdf). Presentations to introduce the project were followed by an open discussion about the project and AT in general. Key stakeholders involved in AT provision including government, non-governmental organisations (NGOs), DPOs and private sector AT suppliers were invited. The launch was followed by meetings with the stakeholders to further gather reactions to and feedback on the project. This space afforded the project leaders with valuable guidance on how to proceed. The launch, development process and roll-out continued throughout 2016 and into 2017 as shown in Figure 1.

During the scoping phase, the project team identified information gaps for each of the major stakeholder groups within the AT sector and evaluated the likelihood of these groups to engage in the project. Achieving the original use case of the application (App), that is, to provide end users of AT with information about the location and availability of AT products, showed low feasibility owing to challenges in engaging the public sector. Given that the majority of end users obtained AT through the public sector, involvement of relevant government ministries was required to gather AT availability information. Therefore, the use case was revised to target public, private and civil society intermediaries who were positioned to purchase directly from AT suppliers and then provide AT to end users. Bridging the information gap between people/groups that are likely to purchase AT and AT suppliers was determined to be the necessary and feasible use case for the App.

In the pilot phase, stakeholders could only obtain the App through SAFOD trainings or outreach. Thirty stakeholders were reached during the first round in April to July 2016, and 40 in November and December 2016. Persons with sensory (hearing, vision and albinism), physical and intellectual and/or developmental impairments were included among stakeholders. The App testing process for both V1 and V2 was started by providing instructions for downloading the App on participants’ phones. Participants would then login on their phone or on a demonstration phone offered by SAFOD and begin navigating through different sections of the App. Depending on the composition of stakeholder groups, instructions were provided on how to enter and save content (portal App) and/or how to view AT suppliers and disability services records (consumer App). Data collected from both AT suppliers and disability service providers included contact information, map information (latitude and longitude) and fields that describe the type of AT or services provided. The project team addressed and documented technical and content questions that emerged during the testing sessions, and asked for specific recommendations for improvements. All feedback was synthesised to inform subsequent App revisions.

The taxonomy used in the App was based on ISO 9999 (2016). The final plain-language categories were informed through an iterative process of testing and user feedback. Eight of the twelve ISO 9999 AT classes with select subclasses were initially included. In response to feedback from DPOs in Botswana, two further subclasses, that is, skin and eye protection (important for people with albinism) and reproduction and sexuality were included. These two aspects reflect critical advocacy areas in southern Africa that are often overlooked. User feedback further showed that the eight ISO 9999 classes were not intuitive/user-friendly enough to constitute first-level categories for the App. However, the 60 subclasses were too many. Thus, a plain language list of 18 categories was created by combining subclasses into broad functional groups as shown in Table 1. The WHO essential assistive devices list of 50 items (WHO 2016) is included in these categories.

**TABLE 1:** The 18 plain-language categories as developed from ISO 9999 classes and subclasses.

ISO 9999 classes	Twenty-three plain-language categories for AT-info-map	Final list of 17 plain-language categories
Orthoses and prostheses (06)	Prostheses and orthoses	Orthoses and prostheses
Assistive products for self-care activities and participation in self-care (09)	Toileting and bathing	Toileting and bathing
	Eye and skin protection	Eye and skin protection
	Reproduction and sexuality	Reproduction and sexuality
Assistive products for activities and participation relating to personal mobility and transportation (12)	Personal mobility	Personal mobility
	Supported transfer	
	Orientation and navigation	Orientation and navigation
Assistive products for domestic activities and participation in domestic life (15)	Eating, drinking and feeding	Eating and drinking
Assistive products for measuring, supporting, training or replacing body functions (04)	Pressure fare items	Pressure care
	Standing frames	Standing, lying and posture
Furnishings, fixtures and other assistive products for supporting activities in indoor and outdoor human-made environments (18)	Sitting and lying support	
	Environmental modifications	Environmental modifications
Communication and information management (22)	Vision enhancement	Vision
	Hearing enhancement	Hearing
	Writing and braille	Writing, reading and braille
	Audio and visual information	Communication
	Face-to-face communication	
	Telephones and messaging	
	Reading	Writing, reading and braille
	Mobile and computer devices	Mobile and computer devices
	Clocks and alarms	Clocks, alarms and memory
	Memory support	
Assistive products for controlling, carrying, moving and handling objects and devices (24)	Reaching, grasping and positioning	Reaching, grasping and positioning

AT, assistive technology.

## Benefits identified

Including persons with disabilities and other stakeholder groups in the design and testing process of the App was seen as positive by participants and SAFOD. Developing a mobile application with specific focus to benefit persons with disabilities, an often neglected minority (Visagie & Swartz 2017), was also hailed as empowering. Persons with disabilities, service providers and representatives from NGOs and DPOs were enthusiastic about the App and indicated that they would use it and would raise further awareness on it among their networks. They felt that the App addressed a gap in their current knowledge and would assist them to identify AT and suppliers in a quick and efficient manner. They enthused about the simplicity of the App and the location feature, which could assist them in determining the proximity of suppliers. They also mentioned that with the App they can order directly from suppliers and thus save the cost of distribution intermediaries. Finally bringing together AT stakeholders through the project has helped to shine light on access to AT in Botswana.

## Obstacles encountered and recommendations

The majority of persons with disabilities are not buying AT directly from suppliers. They are connected to AT through a private, public or civil society sector intermediary. Persons with disabilities, who are often severely affected by unemployment and poverty (Hanass-Hancock et al. 2017), were also less likely to own or have access to smart phones and mobile data, which is costly in Botswana. Apart from data cost, some people could not download the App because they did not have enough space on their phones.

The cost and availability of data may be the largest risk to overall project success and sustainability. Betjeman, Soghoian and Foran (2013) warned against the challenges of using mobile applications in sub-Saharan Africa where communication technology is relatively undeveloped. That CommCare can be accessed offline is a step in the right direction, but further solutions are needed. Data cost will limit the usage and uptake among the most underserved regions and populations, where persons with disability might be most highly represented (Hanass-Hancock et al. 2017). In the final version of the App, AT categories were reduced from 23 to 18, and all product photos and select filtering features were removed, in a bid to keep the data burden low.

Icons were well received and helped communicate the meaning of many categories. However, categories were occasionally perceived as broad and not intuitive. As an example, some participants were unsure what ‘pressure care’ referred to; and some thought ‘eating and drinking’ referred to eateries or restaurants. Decreasing categories to keep the data burden low led to categories being broad. However, categories could also have been perceived as non-intuitive owing to insufficient familiarity with AT.

A general lack of awareness on different types of AT and how AT can benefit users was identified as a major challenge. This project can help raise awareness and increase knowledge through exposure. It exposes people to AT through the scoping visits and training workshops, as the App is tested and rolled out, and through using the App. Furthermore, outreach and training, as well as the development of educational resources (videos, factsheets) for the project website, is suggested to increase general awareness of AT and its impact on function, participation and quality of life.

While collecting updated product-level data was not feasible owing to data burden and human resource demands, both suppliers and consumers requested that this level of information is included. The proposed solution is to develop a web-based database, which is integrated with the App. In the web system, the taxonomy will be expanded by adding another level so that users can find suppliers that offer a desired product type (e.g. crutches), not just AT category (e.g. mobility). More advanced search features, product photos and specifications as well as links to educational resources will also be included.

Central government representatives have expressed support for the project and are allowing employees to participate but were not willing to provide public sector AT supply information for the App. Public AT provision systems are complex, involve multiple government departments, with many points of provision and supply channels that vary by type of AT. As signatories of the UNCRPD, the governments of southern African countries are required to provide persons with disabilities access to appropriate AT and client-centred AT service delivery. The App and the Global Cooperation on Assistive Technology (GATE) initiative could assist the relevant ministries to achieve that requirement.

A final challenge revolves around project sustainability and the long-term covering of operating costs. Staff will be required to maintain the App and App-related activities. Strategies for generating funding through the App such as advertisements or supplier usage fees must be explored.

## Conclusion

While not a complete solution, this App may assist in linking AT users and suppliers in a more efficient manner. The initial plan of developing an App that can be used to track stock in real time was not feasible. However, the simple directory of AT that was developed is of value to AT users, buyers and suppliers and is easier to maintain and therefore more sustainable. The project and App might also assist in the development of a broader and deeper knowledge base on AT in southern Africa. Financial sustainability is a challenge that should be focused on from the very start in planning projects of this nature.

## Footnotes

1 In this paper, persons with disabilities include all those who might need AT such as elderly people and people with various impairments.
